# Selective Laser Melting Strategy for Fabrication of Thin Struts Usable in Lattice Structures

**DOI:** 10.3390/ma11091763

**Published:** 2018-09-18

**Authors:** Radek Vrána, Daniel Koutný, David Paloušek, Libor Pantělejev, Jan Jaroš, Tomáš Zikmund, Jozef Kaiser

**Affiliations:** 1 Brno University of Technology, Faculty of Mechanical Engineering, Institute of Machine and Industrial Design, Technická 2896/2, 616 69 Brno, Czech Republic; Daniel.Koutny@vut.cz (D.K.); David.Palousek@vut.cz (D.P.); Jan.Jaros@vut.cz (J.J.); 2 Brno University of Technology, Faculty of Mechanical Engineering, Institute of Materials Science and Engineering, Technická 2896/2, 616 69 Brno, Czech Republic; Libor.Pantelejev@vut.cz; 3 Brno University of Technology, Central European Institute of technology BUT, Purkyňova 123, 612 00 Brno, Czech Republic; Tomas.Zikmund@vut.cz (T.Z.); Jozef.Kaiser@vut.cz (J.K.)

**Keywords:** selective laser melting (SLM), AlSi10Mg aluminum alloy, scanning strategy, porosity, roughness, contour strategy, melt-pool size, linear energy, input energy

## Abstract

This paper deals with the selective laser melting (SLM) processing strategy for strut-lattice structure production which uses only contour lines and allows the porosity and roughness level to be managed based on combination of the input and linear energy parameters. To evaluate the influence of a laser scanning strategy on material properties and surface roughness a set of experiments was performed. The single welds test was used to find the appropriate processing parameters to achieve continuous welds with known width. Strut samples were used to find a suitable value of weld overlapping and to clarify the influence of input and linear laser energy on the strut porosity and surface roughness. The samples of inclined hollow struts were used to compare the wall thickness with single welds width; the results showed about 25% wider welds in the case of a hollow strut. Using the proposed SLM strategy it is possible to reach a significantly lower porosity and surface roughness of the struts. The best results for struts with an inclination of 35.26° were achieved with 25% track overlapping, input energy in the range from 9 J to 10.5 J and linear energy *E_lin_* from 0.25 to 0.4 J/mm; in particular, the relative density of 99.83% and the surface roughness on the side of the strut of Ra 14.6 μm in an as-built state was achieved.

## 1. Introduction

Nowadays, metallic porous materials such as cellular structures or foams have a wide range of applications. Metal foams are mostly used for energy-absorbing applications or as filling material of the conventionally produced profiles for weight reduction. Their most significant advantage is relatively cheap mass production. However, the regularity and shape of the foam structure cannot be precisely controlled [1,2,3,4,5]. On the other hand, the additively manufactured cellular lattice structures are mostly used for special parts in aerospace, space, cooling or biomedical applications [6,7,8]. The most important advantages of the lattice structure are their shape regularity and a wide range of possible materials that can be used [9,10]. One of the suitable additive technologies for lattice structure production is selective laser melting (SLM).

SLM is a layer-based metal additive technology allowing for rapid fabrication of porotypes and lightweight components with complex geometry [11,12,13,14]. Fabrication, using SLM, is affected by many process parameters which have a significant effect on the final material properties. The main SLM process parameters are laser power (***LP***), laser speed (***LS***), thickness of the applied powder layer, distance between laser tracks, diameter of the laser beam, and scanning direction [15]. Its influence on the final mechanical properties was examined, especially for solid-based production; however, for lattice structures, this has not been well investigated.

Qiu et al. [8] investigated the influence of the laser power (***LP***) and the scanning (***LS***) speed on the diameter, shape and porosity of the struts made of AlSi10Mg material. The linear dependence between the strut diameter and the increasing ***LP*** was found. The authors used ***LS*** of 3500 mm/s while the ***LP*** was changed in the range from 150 W to 400 W. The diameter of strut changed from 260 μm to 500 μm for the nominal diameter of 300 μm. Due to the number of struts in the lattice structure, the mechanical properties can significantly change. The dependence of the main process parameters on the strut porosity was evaluated, but only for one ***LP*** and ***LS*** level. Abele et al. [16] dealt with dimensional accuracy of the strut structure. The authors tested a laser strategy for lattice structure production focused on high dimensional accuracy of very thin struts (d = 0.2 mm). These struts were produced by only one laser path, and therefore the authors investigated primarily the offset of the laser contour paths. The linear energy ***E_lin_*** (J/mm) and laser spot diameter were used as the main parameters. The authors defined the struts’ size limitation as two times laser spot diameter. Leary et al. [17] investigated a manufacturability and surface roughness of the struts with the orientation typical for struts–lattice structures. They found that the surface roughness on the strut down-skin surface is significantly higher due to a heat transfer and sticking of the surrounding powder on the strut down-skin. The surface roughness is strongly dependent on the strut orientation which was also described by other authors [10,16,17]. Yan et al. [10] explained the higher surface roughness on the down-skin surface by the “stair effect” after slicing of the strut to the single layers. This effect increases at a lower strut inclination, where a greater part of the layer is produced directly on the powder. However, a lower height of layer thickness could decrease this effect. Koutný et al. [18] examined the influence of the strut orientation on the strut size. The samples were measured by a 3D optical scanner and evaluated by maximum inscribed cylinders inside the struts. Correction parameters for the struts’ production with the accurate size were proposed.

Yu et al. [19,20] investigated the influence of the laser power and the scanning speed on the width of the single track. The scanning speed was found to be more influential in relation to the final width than the laser power. Samples fabricated with high energy density had a high porosity in the upper layers because the previous layers were re-melted and gas pores moved up to the current layer. Parts with full density were produced with high laser energy density. Wei et al. [21] showed that the weld samples produced in the linear energy density range of 1.5–1.875 J/cm had a continuous scan track with a relatively smooth surface without intertrack pores. Delroisse et al. [22] studied the influence of strut orientation on the microstructure. They found a heterogenous microstructure in case of inclined struts, while the vertical struts had a fully homogenous structure. The differences were explained by worse heat transfer in the bottom zone of the strut caused by strut orientation.

Koutný et al. [18] also examined the influence of the strut orientation on the strut size for samples made of stainless steel (***SS***) material. The results of ***SS*** material were different compared to aluminum alloy; while the samples of ***SS*** material had the diameter smaller than nominal, the samples of aluminum alloy had a larger diameter. Attar et al. [23] examined commercially pure titanium alloy (CP-Ti) from powder with a wide grain size range up to 100 μm. The authors experimentally investigated the SLM process parameter to produce nearly full dense (>99.5%) CP-Ti cylindrical samples with diameter d = 4mm without any post-treatment.

The present study deals with SLM scanning strategy for struts fabrication using concentric contour laser paths in the entire strut cross-section instead of volume strategy [16]. The main aim is to allow for fabrication of struts–lattice structures with expected mechanical and material properties with high repeatability. This is very important for designing components for aerospace or space industries using finite element method (FEM) analysis [24] and topology optimization with great strength to weight ratio. Due to a large number of the struts in the lattice structure, even a low increase in the single strut dimension can significantly change the mechanical properties of the lattice structure [8,25,26,27,28]. Also, the influence of the ***LS*** and ***LP*** process parameters on the struts’ surface roughness and porosity are described.

## 2. Materials and Methods

### 2.1. Metal Powder Analysis

**Figure 1:**
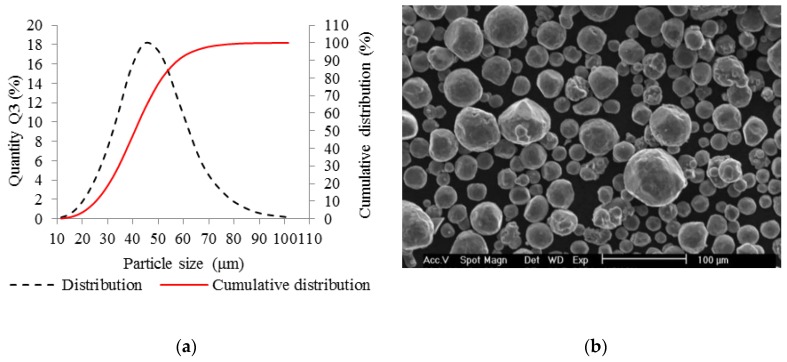
Selective laser melting (SLM) powder characteristics; (**a**) chart of particle size distribution; (**b**) shape of powder particles (scanning electron microscopy (SEM)).

The AlSi10Mg aluminum alloy metal powder (TLS Technik GmbH, Bitterfeld, Germany) was used in all experiments. The metal powder was produced using a gas atomization in nitrogen atmosphere and its particles were almost spherical in shape (Figure 1b). A particle size distribution was analyzed (Horiba LA–960, Horiba, Kyoto, Japan) for powder quality verification. The results can be seen in the chart (Figure 1a). The particle mean size was 41.41 µm, median size was 40.7 µm and standard deviation was 12.9 µm. The particle size up to 25.2 µm represents 10% and the particle size up to 58 µm represents 90% of particle size distribution. Depending on the particle size distribution, a 50 µm layer was applied.

### 2.2. Roughness Analysis

The struts samples were digitized by the optical measurement system (Atos Triple Scan III, GOM GmbH, Braunschweig, Germany) to find out the surface roughness on the strut side. The optical system was equipped with two 8 Mpx cameras and MV60 lens (resolution 17 µm). The samples were coated with a thin layer of TiO_2_ powder (thickness of around 3 µm) [29] before the scanning process and digitized separately one by one for a more detailed measurement. After digitization, the data were polygonised using a “more detailed” option in GOM Atos software. With the optical measurement, the down-skin surface cannot be sufficiently digitized. Therefore, data of micro-computed tomography were used.

The GOM Inspect software was employed to evaluate the surface roughness by comparing the section line of digitized strut surface and the best-fitted computer-aided design (CAD). (Figure 3a). Obtained values were used for calculation of the Ra surface roughness according to Equation (1).

(1) $$Ra\;=\frac{1}{n}\sum_{i=1}^{n}\left|z_{i}\right|=\frac{\left|z_{1}\right|+\left|z_{2}\right|+\ldots +\left|z_{n}\right|}{n}\;\left(\mu m\right)\;$$

### 2.3. Porosity Analysis

**Figure 2:**
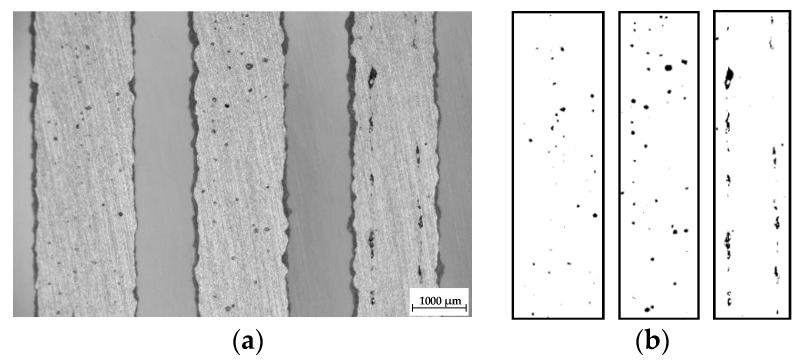
The samples after basic grinding to the struts mid-plane; (**a**) the top view images captured by light microscope; (**b**) three areas of the struts after converting of the colors in ImageJ software.

Software Image J was used for the initial porosity analysis of the top view sample images (8-bites) after basic grinding with the use a hand grinder (GP-2 Grinder, Sinowon, Dongguan, China). The image area for the analysis was cropped out using the rectangle window without inclusion of the rough border of the strut. Then, the colors were converted to black and white using the automatic threshold to reach a repeatability for all samples. The results of the porosity were evaluated as the percentage of black in the color white (Figure 2).

**Figure 3:**
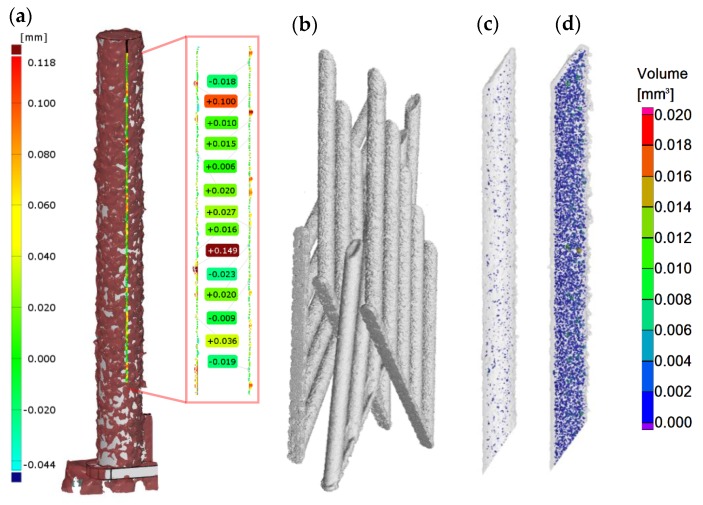
Struts analysis—(**a**) surface roughness evaluation in GOM Inspect software after optical measurement; (**b**) group of four samples measured together in VGStudio MAX software; (**c**) transparent 3D render of the strut with the lowest porosity 0.17% (**d**) transparent 3D render of the strut with the highest porosity 2.93%.

Internal porosity was also analyzed using the micro-computed tomography (µCT, GE phoenix v|tome|x L240, GE, Wunstorf, Germany). The main parameters of the X-ray tube used were the voltage of 130 kV, current of 100 µA, and filter of 0.5 mm copper plate. Within two µCT measurements, two groups of four samples were jointly analyzed (Figure 3b). The measured data were obtained with the 15 µm linear voxel size resolution and were reconstructed (using the back-projection algorithm) in the Datos reconstruction software. All subsequent post-processing was performed in the software VGStudio MAX 3.1.

During the software analysis, the reconstructed data were divided into single struts and then each sample was independently analyzed in the porosity analysis module. The software detection of the air pores is based on the thresholding method that determines the boundary between the material and the air (background). This threshold was calculated automatically by software to reach comparability between both measurements. The results of the porosity analysis were between 0.17 and 2.93% (Figure 3c,d).

### 2.4. Input Energy Calculation

The input energy to the current layer (***E_in_***) was obtained by Equation (2). It is based on the real laser paths in the actual layer, and on beam compensation and hatch distance parameters, which depended on actual process parameters and their single welds. The total length of the laser paths in the layer ***l*** was calculated based on the ellipse circumference ***o*** and the numbers of laser tracks ***N*** (Equation (3), Table 1), ***LS*** and ***LP*** were the main laser parameters.

(2) $$E_{\mathrm{in}}=\frac{\mathrm{LP}}{\mathrm{LS}}\cdot (J)$$

(3) $$\;l=\sum_{i=1}^{n}o_{1}+o_{2}+\ldots +o_{n}\;(\mathrm{mm})$$

(4) $$\;o\;\approx \frac{\pi }{2}\left[a+b+\sqrt{2\left(a^{2}+b^{2}\right)}\right]\;(\mathrm{mm})$$

(5) $$\;a=\frac{d}{2};\;b=\left(\frac{d}{2}\right)\cdot \cos\;\left(54,74^{\circ }\right)\;(\mathrm{mm})$$

### 2.5. Samples Fabrication

All samples were manufactured using a SLM machine (SLM 280^HL^, Lübeck, Germany) equipped with 400 W Ytterbium fiber lasers (YLR) laser. The laser beam was focused to the diameter of 82 µm and had a Gaussian shape. Laser scanning speed may reach up to 10,000 mm/s. During the production process, N_2_ atmosphere was used in the chamber and the oxygen level was kept under 0.2%. The platform temperature was 150 °C.

To find the most suitable material and surface properties of AlSi10Mg struts produced by SLM, several tests were used:

- Single welds test;
- Struts test;
- Struts test II;
- Hollow struts test.

#### 2.5.1. Single Welds Test

The aim of the single welds experiment was to find a suitable combination of the main process parameters (***LP***, ***LS***) for the production of consistent single welds and to find out the width of single welds for a specific combination of process parameters. 

**Figure 4:**
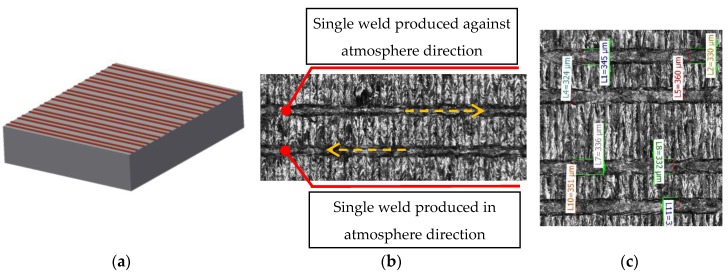
(**a**) Shape of the single welds samples; (**b**) one combination of process parameters produced in and against the atmosphere flow; (**c**) measurement of width of the single welds.

To prepare the real condition during layer by layer production, single welds were produced on the top of 5 mm solid material block (Figure 4a). The influence of the laser direction on the single welds condition was also observed; therefore, all single welds were produced in and against atmosphere flow direction (Figure 4b). Images of the welds from the top view were captured by light microscope (Olympus SZX7, Olympus, Tokyo, Japan) and used for width measuring and a visual evaluation of the continuity and uniformity (Figure 4c). Their width was measured in six points along each single weld and one average value for both directions was used. For the experiment, the following process parameters were changed—***LP*** in the range between 175 and 400 W in steps 25 W and ***LS*** in the range between 200 and 2000 mm/s in steps 100 mm/s.

#### 2.5.2. Strut Test

**Figure 5:**
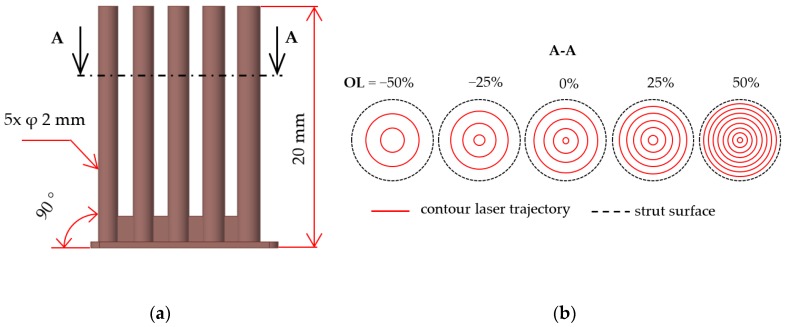
(**a**) Shape of the samples with inclination of 90°; (**b**) contour strategy with different overlap parameter.

The aim of the test was to narrow the process parameter window of the single weld test depending on the porosity and surface roughness of the struts and to find the most suitable overlap (***OL***) parameter from the porosity point of view. The samples consisted of five struts with diameter d = 2 mm were produced in two orientations (***OR***) compared to the platform ***OR*** 90 and ***OR*** 35.26°. The strut diameter was chosen to be sufficiently large to set ***OL*** in the range from ***OL***-50% to ***OL*** 50% of the weld width (Figure 5b). The beam compensation parameter (***BC***, a distance between the strut surface and the first laser path) was applied as a half of the weld width. Laser process parameters were changed as follows—***LP*** in the range from 225 to 350 W, and ***LS*** in the range from 400 to 2000 mm/s.

In this experiment, the laser strategy for lattice structure production created only by contour lines was tested (Table 1). The main idea is the possibility to produce fine lattice structure using various combinations of the process parameters (low/high energy) to manage surface roughness or internal porosity and to allow for production of very thin struts. An advantage is an easy optimization of the strategy for the elimination of non-melted or over-melted areas in struts and for improving dimension accuracy.

After fabrication, samples were ground to the mid-plane of the struts using a hand metallographic grinder (GP-2 Grinder, Sinowon, Dongguan, China), and the images of the samples from the top view were taken by light microscope (Olympus SZX7, Tokyo, Japan). For the initial porosity analysis ImageJ software was used. Porosity was analyzed only on the samples with ***OL*** 0%, 25% and 50%.

#### 2.5.3. Strut Test II

The aim of the experiment was to find the influence of ***LP*** and ***LS*** on the internal porosity and surface roughness. The following process parameters were selected: ***LP*** in the range between 225 W and 400 W and ***LS*** in the range between 500 mm/s and 2000 mm/s with respect to the perspective area of previous strut test. The strut samples with ***OR*** 35.26° only were used in the experiment. The samples were analyzed using µCT to obtain more accurate results of porosity and full surface data for down-skin roughness evaluation.

#### 2.5.4. Hollow Struts Test

**Figure 6:**
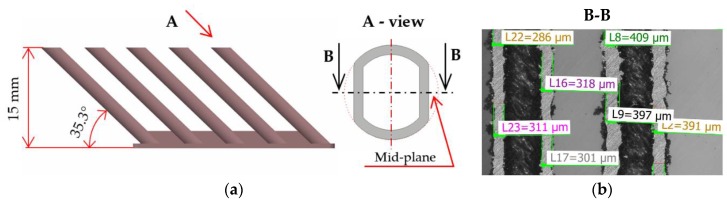
(**a**) A special shape of strut designed for evaluation of the width of hollow strut walls without distortion caused by inclined grinding plane; (**b**) wall width measurement using macro images captured by the light microscope.

Samples of hollow strut shape; created with only one single weld in each layer, were produced (Figure 6). The cross-section of the hollow strut sample was designed to ensure evaluation of correct width of the wall without distortion caused by grinding in the inclined plane. The primary aim of the hollow strut test was to compare the width of the single welds on the solid block and the width of the wall of hollow struts (the shape close to the real strut). The combinations of the process parameters were selected also to obtain the influence of the width of wall on ***LP*** and ***LS***. For this experiment, the following process parameters were changed—***LP*** (225, 250, 275, 300, 350, 400 W); ***LS*** (500, 900, 1400 mm/s). After fabrication, the 37 samples were ground to the mid-plane of the struts using a hand metallographic grinder (GP-2 Grinder, Sinowon, Dongguan, China) and the images of the ground surface from the top view were captured by light microscope (Olympus SZX7, Olympus, Tokyo, Japan).

## 3. Results

### 3.1. Single Welds Test

**Figure 7:**
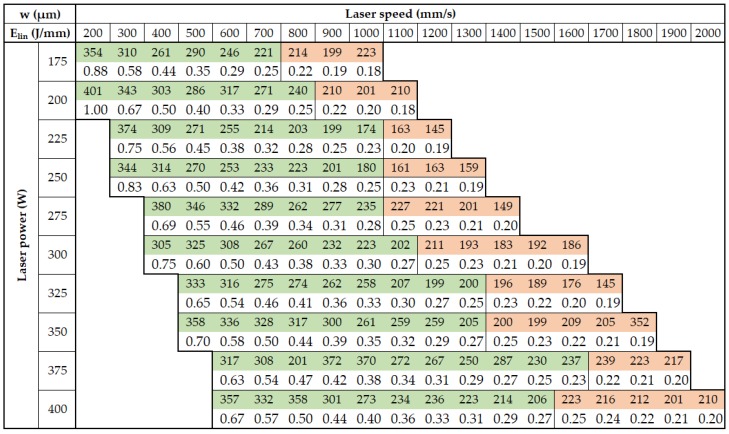
The average width of the single welds in and against atmosphere flow (colored cells); line energy (color free cells).

The results of the width of single welds are shown in Figure 7. The final values were averaged from six measurements against and six measurements in the atmosphere flow direction. The values marked in red color were excluded due the worse quality of the welds (non-uniformity of width and bad continuity). For the input linear energy calculation, Equation (6) was used and the limit value of around 0.25 J/mm was found for continuous welds.

(6) $$\;E_{lin}\;=\;LP/LS\;\left(J/\mathrm{mm}\right)$$

**Figure 8:**
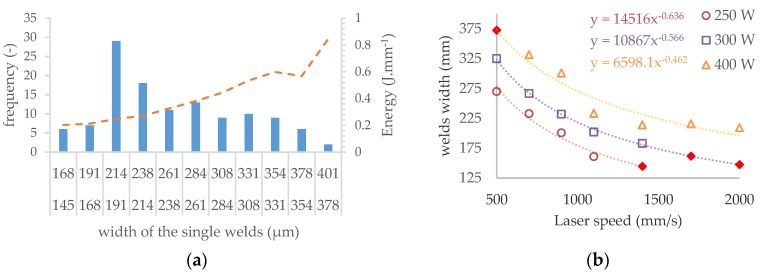
The results of the single welds test (**a**) histogram of the width frequency of single welds, (**b**) the prediction of weld width for non-tested combinations of process parameters.

Figure 8a shows the frequency of the continuous single welds widths from 145 µm to 401 µm in all tested process window. Different widths of single welds are useful for ensuring the dimension accuracy and material properties during production of the struts using a contour line strategy (especially thin struts). Therefore, for the next experiments, the welds across the entire perspective process window were selected as follows—the consistent welds were categorized into 11 classes according to weld widths. From each class, one combination of process parameters was chosen depending on the amount of linear energy ***E_lin_*** in the (Equation (6)). The combination of ***LP*** and ***LS*** with the linear energy level closest to the average energy level of the class was selected. A few more combinations, e.g., laser parameters corresponding with standard process parameters or the parameters from previous studies, were chosen. Finally, 16 combinations of ***LS*** and ***LP*** were tested.

### 3.2. Struts Test

#### 3.2.1. Determining the Overlap Parameter

**Figure 9:**
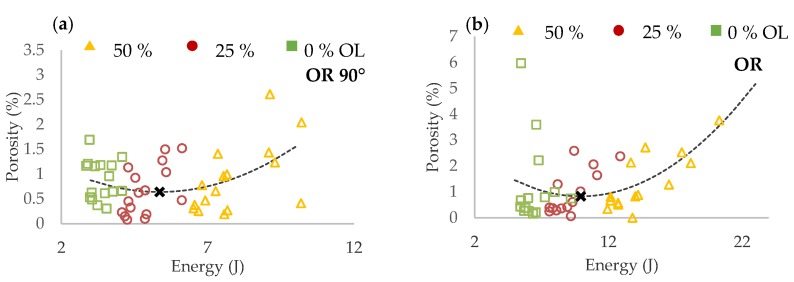
Porosity vs. input energy dependence (**a**) for inclination 90°; (**b**) for inclination 35.26°.

The samples were ground to the mid-plane of the struts to measure the internal porosity and to check the weld overlap (***OL***). The internal porosity was analyzed on the struts with ***OL*** from 50% to 0% to prevent distortion of the results due to a disconnection between the neighboring single welds. For evaluation of the most suitable ***OL*** value, a dependence of the porosity vs. input energy ***E_in_*** was used (Figure 9a,b).

In the charts, three groups of porosity regarding the ***OL*** parameter were identified in both cases of struts inclination. To find out the ***OL*** parameter with the lowest porosity level, the results were interpolated with quadratic polynomial function and the minimum of the function was determined (black cross in Figure 9).

In the results of both strut inclination, the overlap ***OL*** 50% shows a higher porosity level. It occurs due to a large overlap area where the material is overheated. Higher porosity level is also at ***OL*** 0% where it could be caused by theoretically no overlap and insufficient connection between the neighboring welds. The lowest porosity level was reached at ***OL*** 25% in both orientations; therefore, for the next experiments, the ***OL*** 25% was selected as the most suitable. In the case of ***OR*** 35.26°, higher porosity values were identified compared to ***OR*** 90°.

#### 3.2.2. Initial Roughness Analysis

**Figure 10:**
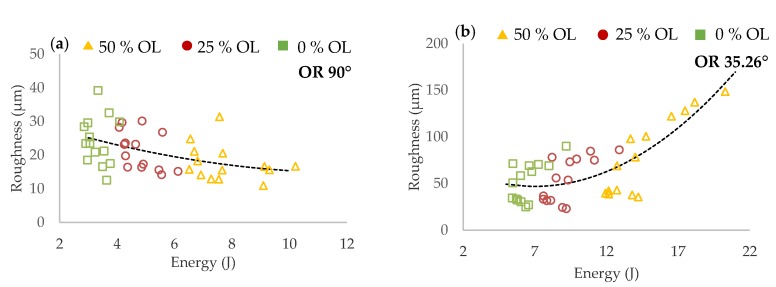
Surface roughness vs. input energy dependence (**a**) for inclination of 90°; (**b**) for inclination of 35.26°.

Results of surface roughness on the strut side show different trends based on struts inclination (Figure 10). For the struts of ***OR*** 90°, roughness decreases with higher ***E_in_*** while for ***OR*** 35.26°, roughness significantly increases with higher ***E_in_***. The worse surface quality at ***OR*** 35.26° is caused by approximately 40% higher ***E_in_*** and heat transfer to the surrounding powder particles and caused by strut inclination.

**Figure 11:**
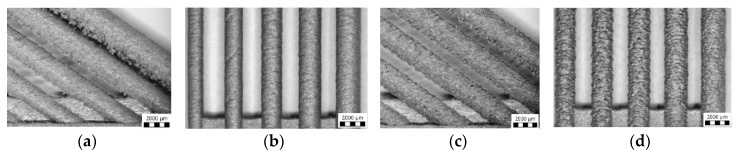
Struts surface quality—produced with higher ***E_in_*** (**a**) at orientation (***OR)*** 36.26°; (**b**) at ***OR*** 90°; produced with lower ***E_in_*** (**c**) at ***OR*** 36.26°; (**d**) at ***OR*** 90°.

The images captured by light microscope (Olympus SZX7, Tokyo, Japan) confirm the previous results. Figure 11 shows two combinations of process parameter. The former with high ***E_in_***–***LP*** 275 W, ***LS*** 400 mm/s (Figure 11a,b), and the latter with low ***E_in_****–**LP*** 300 W, ***LS*** 1400 mm/s (Figure 11c,d). In the case of the struts at ***OR*** 90° produced with higher ***E_in_***, the surface quality was smooth, with no partially melted powder (Figure 11b). The struts produced with lower ***E_in_*** were characterized by visually rough struts surface (Figure 11d). In the case of the struts at ***OR*** 35.26° produced with higher ***E_in_***, the top surface also seems to be smooth; however, a lot of partially melted powder appeared on the strut down-skin surface (Figure 11a). The struts produced with lower ***E_in_*** had a significantly smaller amount of partially melted powder on the strut down-skin surface (Figure 11c).

The results of porosity and surface roughness were jointly used to narrow the perspective area of the process parameters for struts production in 3D contour graph (Statistica software). Based on the results, the laser parameters were narrowed as follows—***LP*** between 225 W and 300 W, ***LS*** over 1000 mm/s.

### 3.3. Struts Test II

#### 3.3.1. Interpolation of Welds Width

The aim of the struts test II mainly was to determining the effects of individual parameters on surface roughness and internal porosity. In order to obtain dependencies on ***LP*** and ***LS***, the parameters were selected with respect to perspective area of the previous strut test. Some combinations of ***LP*** and ***LS*** were not included in the single welds test and their widths were not known. To find them, the dependence between ***LS*** and width of the single weld was used for prediction (Figure 8b).

Figure 8b shows the curves of ***LS*** vs. welds width curves for ***LP*** 400, 300 and 250 W. From the results of ***LP*** 400 W, it is obvious that the dependence is not linear; therefore, the power functions, which best correspond to the measured data, were used for interpolation. This is also confirmed by the results for ***LP*** 300 W and ***LP*** 350 W. The calculated values are marked red.

#### 3.3.2. Porosity Analysis

The porosity results were obtained by a micro-computed tomography (µCT) device with voxel size of 15 µm. The struts were individually analyzed to find out the porosity level for each combination of process parameters However, the shape and the size of pores is also important. Table 2 shows the images of the µCT analysis with various shapes of pores and porosity level. Based on the results, the required minimum values of linear energy ***E_lin_*** 0.25 J/mm and input energy ***E_in_*** 8 J were identified for strut production without creating large irregular pores.

#### 3.3.3. Evaluation of Perspective Laser Parameters

**Figure 12:**
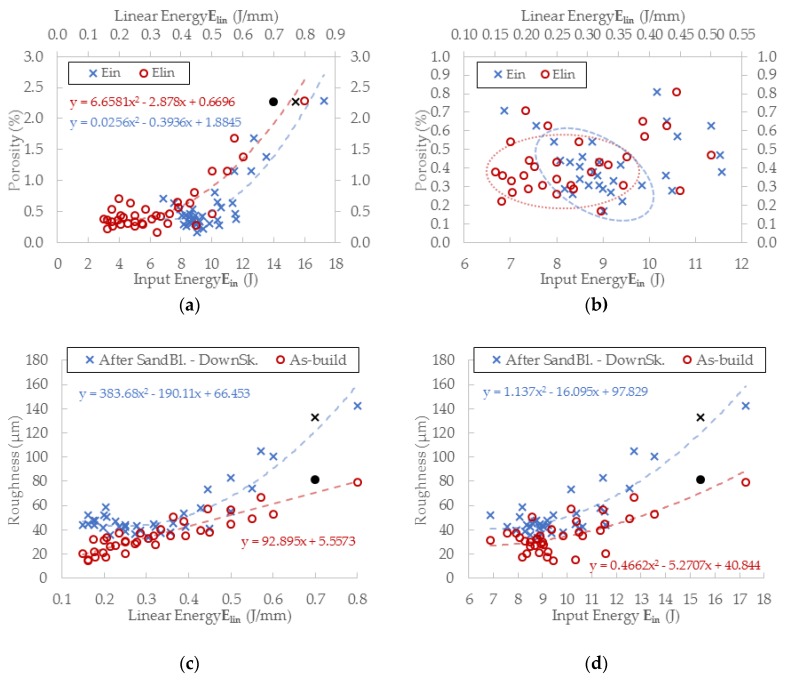
(**a**) µCT porosity vs. input and linear energy dependences, black mark represents the universal SLM process parameters; (**b**) focused results area up to 1% porosity level with marked perspective areas; (**c**) surface roughness vs. linear energy dependence—on the side and down-skin struts surface, black marks represent the universal SLM process parameters.; (**d**) surface roughness vs. input energy dependence.

**Figure 13:**
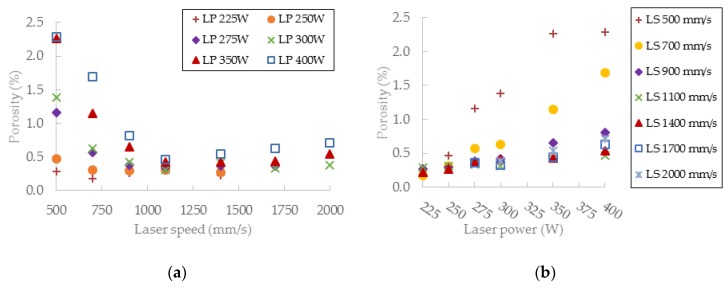

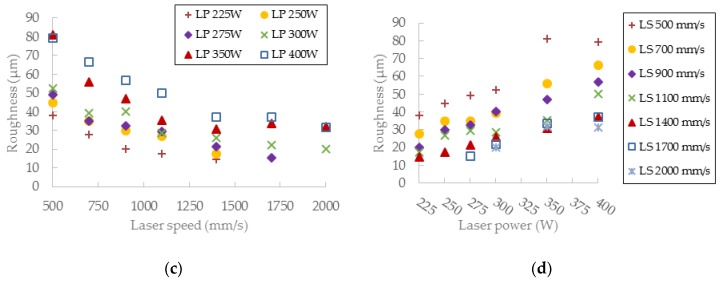
(**a**) The influence of various laser speed (LS) levels on porosity; (**b**) the influence of various laser power (***LP)*** levels on porosity; (**c**) the influence of various LS levels on surface roughness; (**d**) the influence of various ***LP*** levels on surface roughness.

The results of porosity levels generally show a similar trend as in the initial results of the previous test. However, in this case, the significant accumulation of results at the porosity level 0.4% occurs for ***E_in_*** of 8 ÷ 10 J and for ***E_lin_*** of 0.15 ÷ 0.4 J/mm (Figure 12a,b). However, the porosity level of linear energy ***E_lin_*** in range 0.15 ÷ 0.25 J/mm is very low, and the porosity is created with a small number of large irregular pores. It can significantly decrease the mechanical properties; therefore, this area is unsuitable for the production of the struts. Regarding the charts, which show the influence of ***LS*** and ***LP*** on the porosity (Figure 13a,b) and the previous porosity analysis, the parameters ***LP*** of 225 ÷ 275 W, ***LS*** of 900 ÷ 1400 mm/s with ***E_in_*** of 8 ÷ 10.5 J, ***E_lin_*** of 0.25 ÷ 0.4 J/mm, and ***OL*** 25% were selected as the perspective for struts production from the porosity point of view.

The results of surface roughness were obtained by µCT measurement in this experiment; therefore, it was possible to analyze the results on the side and also down-skin strut surface (Figure 12c,d). The results show a similar trend as the results of porosity except for the pronounced linear dependence of as-built surface roughness on linear energy ***E_lin_***. The best results were accumulated between ***E_in_*** 8 ÷ 10 J and ***E_lin_*** of 0.15 ÷ 0.4 J/mm with the level of about Ra 30 µm on the strut-side surface and about Ra 40 µm on the down-skin surface.

Regarding the charts, which show the influence of LS and LP on the roughness (Figure 13c,d), parameters LP in the range of 225 ÷ 300 W, LS in the range of 900 ÷ 2000 mm/s with Ein of 8 ÷ 10 J, Elin of 0.15 ÷ 0.4 J/mm and OL 25% were selected as the perspective for lattice structure production with a low roughness level.

### 3.4. Wall Width Analysis

**Figure 14:**
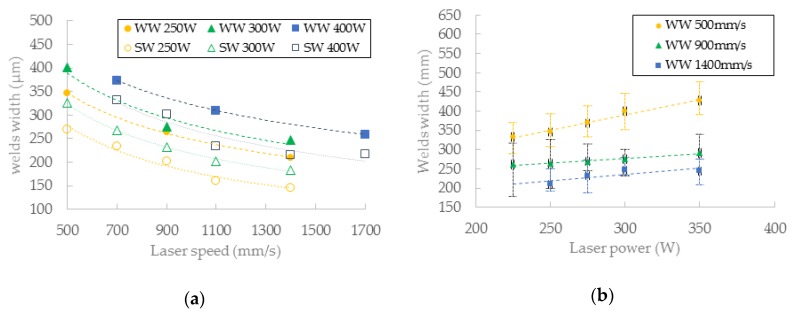
(**a**) Comparison of single welds and walls width according to ***LS***—in chart (SW) single weld width; (WW) wall width; (**b**) the wall width according to ***LP***.

Simultaneously with the strut samples, hollow strut samples were fabricated. The hollow strut shape was used because of similar heat transfer conditions as those in the case of struts production. The width of the wall at ***OR*** 35.26° was of about 25% higher on average than that in the case of the single welds on a solid block (Figure 14). The results confirm the trends of the weld widths from a single weld test. The influence of ***LP*** seems to be more linear than that of ***LS***.

### 3.5. Metallographic Analysis

**Figure 15:**
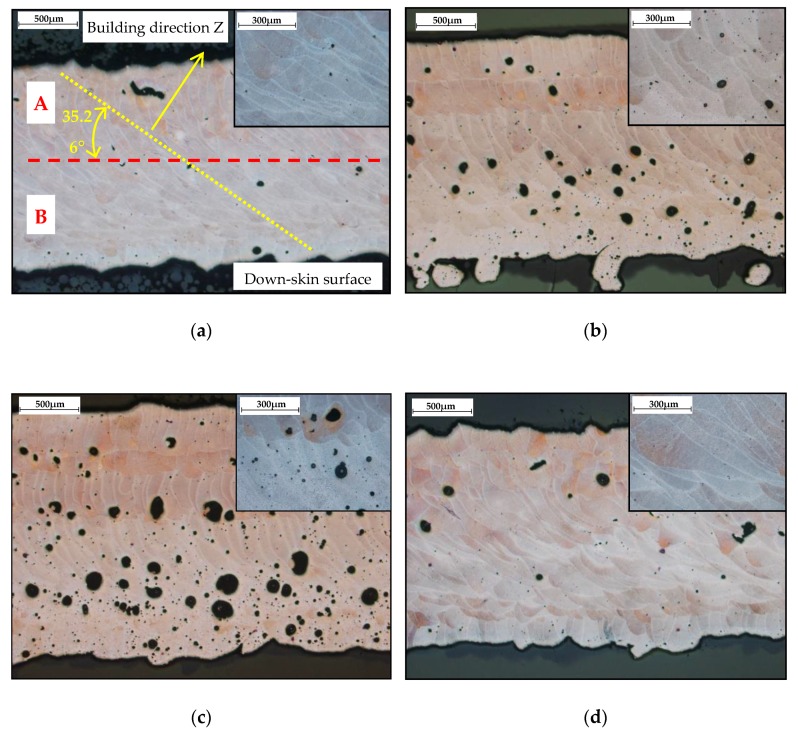
Microstructure of the struts (**a**) ***LP*** 250 W, ***LS*** 1400 mm/s, ***E_in_*** 9.17 J, ***E_lin_*** 0.18 J/mm with description common for all pictures; (**b**) ***LP*** 300 W, ***LS*** 500 mm/s, ***E_in_*** 13.54 J, ***E_lin_*** 0.6 J/mm (**c**) ***LP*** 350 W, ***LS*** 500 mm/s, ***E_in_*** 15.43 J, ***E_lin_*** 0.7 J/mm; (**d**) ***LP*** 400 W, ***LS*** 1700 mm/s, ***E_in_*** 7.56 J, ***E_lin_*** 0.24 J/mm.

A metallographic analysis for evaluation of the microstructure was performed. Standard methods were used for metallographic sample preparation, i.e., wet grinding and polishing with use of diamond pastes. A microstructure of the struts was analyzed in an etched state (Fuss etchant) and evaluated by metallographic light microscope (Olympus GX 51, Tokyo, Japan). Orientation of the micrographs is parallel to the strut axis (Figure 15a). The microstructure of the struts is inhomogeneous, consisting of single welds separated by fusion boundaries. Differences in the microstructure can be seen in the layers close to the down-skin surface of the struts (B area in Figure 15a) in comparison with the up-skin surface (A area). Different shapes of porosity depend on the ***E_in_*** parameter. Due to overheating of the material, gas pores with a spherical shape were created (Figure 15b,c). No cracks were found in the microstructure of the evaluated samples.

## 4. Discussion

### 4.1. Comparison of the Linear Energy Needed for Consistent Single Weld

The results of the single welds experiment show as a limit value ***E_lin_*** 0.25 J/mm for consistent welds. The value is higher than that in the case of [21], where the determined limit was 1.5 J/cm. The difference is caused by the shape of the used sample. In [21], the single welds were fabricated directly on the platform. In the present study, the sample, which simulates real production and the increase of thickness of the deposited powder during the first few layers, was used. After melting, the produced layer has a height usually lower than that of the originally spread layer of 50 µm. Then, the next deposited layer has higher thickness and a quality of weld and the required linear energy ***E_lin_*** can be changed [30].

### 4.2. Benefits of Contour Lines Laser Strategy

**Figure 16:**
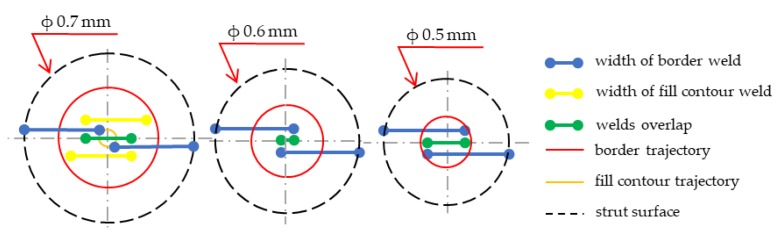
SLM Solutions universal struts laser strategy.

The state that led to the design of a contour scanning strategy is shown in Figure 16. There are examples of the laser strategy internally developed by SLM Solutions universal process parameters. In the cases of diameters of 0.5 and 0.6 mm, only one contour line was generated while in the case of 0.7 mm diameter, one more fill contour line was generated. Using the results of the single weld test, it is possible to calculate the theoretical dimensional accuracy and re-melting area.

The laser parameters of universal process parameters are as follows: contour line ***LP*** 350 W, ***LS*** 500 mm/s; fill contour offset ***LP*** 250 W, ***LS*** 555 mm/s, and ***LF*** = −4). During production of a 0.5 mm strut, the diameter will be theoretically about 0.116 mm (23%) larger because of a combination of the beam compensation parameter of 0.15 mm and the weld width of 0.358 mm. A theoretical overlap area is 0.158 mm (44% of the weld width). In the case of a 0.6 mm strut, the diameter will also be larger (about d = 0.116mm; 19%) and a theoretical overlap area is 0.058 mm (16% of the weld width). The diameter of 0.7 mm will also be about 0.116 mm larger; as can be seen in Figure 16, from the contour line, no overlap area is created. Therefore, a fill contour track in the distance of 0.17 mm from the contour line is added. Due to the fill contour line and its distance from the contour path, an unfavorable state with large overlap of 0.31 mm (87% of the weld width) is created in the center of the strut. Its trajectory is also on the already produced contour weld and thus the material is re-melted, which can cause internal defects in the struts (Figure 16; d = 0.7 mm). The aim of the proposed contour strategy is to create uniform conditions for different strut diameters and improve the dimension accuracy using single welds results.

Using the previous results of the single welds experiment, the hollow strut experiment and a designed script in Excel, the contour strategy for production of the struts with low porosity, and surface roughness, were designed (Table 3). The obtained weld widths from the single weld experiment which meet the required linear energy ***E_lin_*** and input energy ***E_in_*** were used only (Figure 19a). These values were increased by about 25% (parameter from the hollow strut test, Figure 19b) and then used to define the beam compensation ***BC*** parameter ((single width × 1.25)/2). The goal of the Excel script was to find a suitable combination of laser parameters which achieve the overlap in the center of the struts as close as possible to the value around ***OL*** 25%. The results are shown in Table 3. For the diameters of 0.5 and 0.6 mm, the combinations with the required ***OL*** parameter have been found. In the case of the diameter 0.7 mm, the best results were obtained with ***LP*** 225 W, ***LS*** 900 mm/s and ***LP*** 250 W, ***LS*** 1000 mm/s, however the ***OL*** parameter between neighboring welds must increase to 29% and 34%. For values closer to ***OL*** 25%, it would be necessary to discover other combinations of parameters around these two; however, the expected levels of porosity and roughness using these combinations will be significantly lower compared to standard SLM strategy (Figure 12).

### 4.3. The Heat Transfer during Strut Fabrication

**Figure 17:**
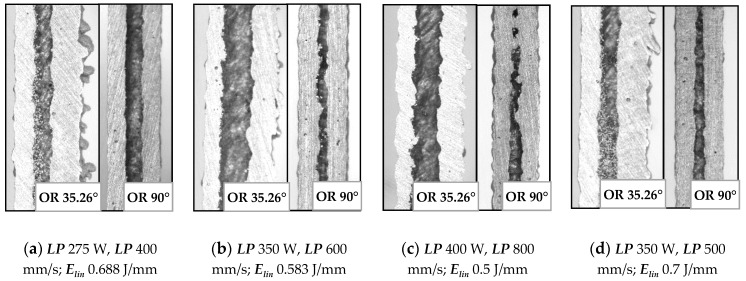
Comparison of inclined and perpendicular strut (**a**) for parameters ***LP*** 275 W, ***LP*** 400 mm/s; (**b**) ***LP*** 350 W, ***LP*** 600 mm/s; (**c**) ***LP*** 400 W, ***LP*** 800 mm/s; (**d**) ***LP*** 350 W, ***LP*** 500 mm/s.

**Figure 18:**
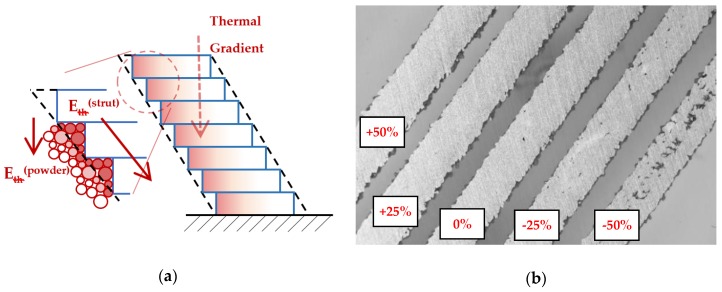
(**a**) Scheme of the heat transfer during SLM additive manufacturing; (**b**) The ground sample to the mid-plane of the struts.

The first strut experiment in this study was also designed for comparison of the conditions during production the struts with ***OR*** 90°and ***OR*** 35.26°. The difference is caused by worse heat transfer in the inclined strut. It can lead to wider single welds than expected; therefore, the successful results of porosity at ***OL*** 0% were discovered (Figure 9, Figure 18b). This was verified by the hollow struts experiment which confirmed this hypothesis. The width of the wall was increased on average by about 25% (Figure 14a). At higher energy levels (over 0.5 J/mm), the effect of the wider bottom part of the wall also appeared (Figure 17). It was caused by the thermal gradient during SLM production of struts with inclination and shows the heat energy transfer well. To describe the energy conditions during the struts’ production, this must be considered:

(1) Due to the point contact of the powder particles between themselves, the metal powder has much lower heat conductive performance and works as an insulator compared to the solid material.
(2) Due to the strut inclination, the cross-section with a higher area occurs in every layer. Using the energy calculation in Equation (2), it is possible to calculate the increase of the input energy ***E_in_*** and compare ***OR*** 35.26° and ***OR*** 90°; it is about 40% higher in the case of ***OR*** 35.26°.
(3) The thermal gradient points in the direction **-*Z***. Due to the inclination of the struts, the heat transfer is slower than in the case of the strut with the axis directed in thermal gradient direction.

After melting of each layer of the strut, the heat energy flows straight down in thermal gradient direction. There are two parts of each produced layer with different energy transmission, the part produced on the previous layer and overhanging part produced on the powder (Figure 18). In the former case of the part on the previous layer, the energy flows through the strut. Because the thermal gradient has a different direction than the strut, heat transfer is slower compared to the strut with ***OR*** 90°.

In the latter, the thermal energy flows to the powder which is in contact with the strut down-skin surface and the powder particles are overheated because there is only a point contact between neighboring powder particles, and only poor heat transmission to the powder bed. Affected particles are melted on the down-skin surface which causes larger dimensions of the struts [8,18] or a wider bottom part of the hollow struts samples, as well as a higher surface roughness on the down-skin surface of the strut (Figure 17). The heat energy is also accumulated in overhanging part of the layer and the material structure is changed [22] (Figure 15).

### 4.4. Porosity and Roughness Analysis

The results of µCT show a different shape and level of porosity according to the input energy ***E_in_*** and the linear energy ***E_lin_***. The porosity in the struts with the lowest porosity level is often formed by a small number of larger pores which are located close to the top surface (Table 2). Formation of the large irregular pores is related to the heat transfer in the inclined struts where more heat energy is accumulated at the bottom part of the strut. Due to the low linear energy, there is no overheating area in the bottom part of the strut (the spherical porosity is very small); however, on the upper side, the state is an unstable because of a lack of linear energy. This causes the occasional disconnection of neighboring welds and formation of larger pores. The minimum value ***E_lin_*** 0.25 J/mm was determined; it is in line with the results of single welds.

A lack of ***E_in_*** causes porosity in the center of the struts. During production of the laser tracks close to the strut surface, heat transfer is lower due to the surrounding powder. During production of single welds in the center of the strut, heat transfer is higher due to the neighboring welds; an unstable state occurs with occasional disconnection of the welds and formation of larger pores. The minimum value ***E_in_*** 9 J was determined. Also, the inside-out order of single welds production is recommended.

### 4.5. Porosity and Roughness

The current results of strut experiments clearly show that the porosity and surface roughness is affected by the input energy ***E_in_*** as well as linear energy ***E_lin_***; these both include the laser power ***LP*** and the laser speed ***LS***. It follows that for strut production free of internal defects and a rough surface, appropriate laser process parameters must be chosen. The charts of the dependences of ***LP*** and ***LS*** on the porosity (Figure 13) demonstrate a different influence of the parameters on porosity forming. The chart shows that the porosity increases with higher ***LP*** at all ***LS*** levels linearly, except for ***LS*** level of 500 mm/s, which shows unstable results. The ***LS*** parameter shows non-linear results with the lowest porosity in the range of 1000 ÷ 1250 mm/s. With higher ***LS,*** the porosity seems to be stable and at the constant level, except for ***LP*** 400W where the porosity increases. ***LSs*** up to 1000 mm/s are unstable and the porosity significantly increases. It could be caused by too high ***E_in_*** and formation of gas pores in the material due to its overheating material (Figure 15c).

The influence of ***LS*** and ***LP*** on the surface roughness has a similar character as that on the porosity in case of input energy ***E_in_***. Linear energy ***E_lin_*** dependence shows the pronounced linear dependence of as-built surface roughness. In Figure 13c, it is possible to find two approximately linear areas. The first area, up to 1400 mm/s, where there are significant changes in roughness values, and the other one, between 1400 mm/s and 2000 mm/s, where there is a significantly lower and stable roughness level. The dependence of ***LP*** vs. roughness is linear for all tested laser speeds. Generally, the results show a low surface roughness with lower ***E_in_*** and ***E_lin_***.

## 5. Conclusions

**Figure 19:**
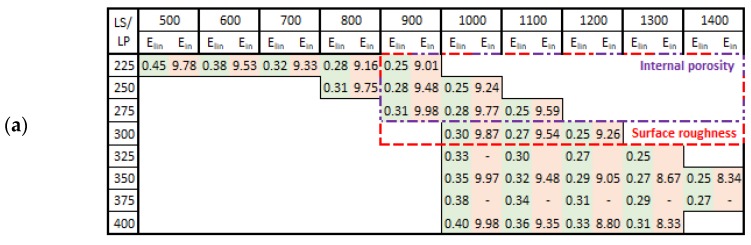

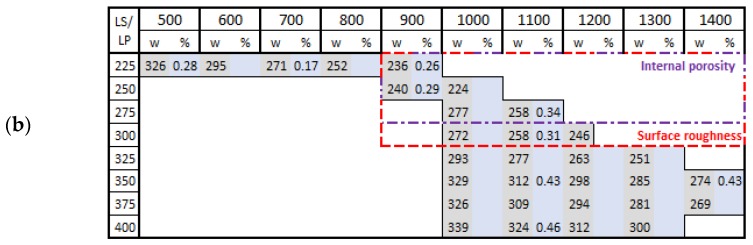
The perspective area (**a**) linear energy ***E_lin_*** (J/mm) (green cells) and input energy to the layer ***E_in_*** (J) (red cells); (**b**) width of single welds in (µm) multiplied × 1.25 (parameter from hollow strut test) (grey cells) and porosity level (blue cells).

In this article, an experimental study was conducted to investigate the influence of a proposed contour line laser strategy for an SLM lattice structure on internal porosity and surface roughness of the single struts, which significantly affects the mechanical properties of the lattice structure. Based on the dependence of porosity vs. input ***E_in_*** and linear energy ***E_lin_***, the influence of the laser speed ***LS*** and laser power ***LP*** was found and the perspective areas of suitable process parameters for the struts–lattice structure were defined. In the present study, the main conclusions are as follows:

- For the production of the struts–lattice structure, the contour strategy seems to be perspective, mainly because of the possibility to use various laser process combinations based on the required width of single welds of the different strut dimensions to achieve a fully melted strut with a constant ***OL*** 25% parameter.
- The heat transfer condition in the inclined struts significantly influences all material and shape parameters of the struts (lattice structure). During the strut production with high ***E_in_***, heat energy is accumulated in the down-skin part of the strut and higher roughness, higher porosity and change of the material microstructure occur. Therefore, the production at lower ***E_in_*** levels leads to more stable results with lower porosity and roughness.
- ***E_in_*** calculated based on the real laser trajectory in the strut describes the amount of the porosity (P) and roughness (R) in the strut samples (d = 2 mm) well. Another necessary condition for struts production without large and irregular internal pores is the minimum level of linear energy ***E_lin_*** 0.25 J/mm. The perspective areas of process parameters based on ***P*** and ***R*** were defined as follows—***E_in_*** of 8 ÷ 10 J; ***E_lin_*** of 0.25 ÷ 0.4 J/mm, ***LP*** of 225 ÷ 300 W, ***LS*** of 1250 ÷ 1750 mm/s and ***OL*** 20% ÷ 30%. Figure 19 shows the perspective area which meets all conditions for low porosity and surface roughness levels. The presented weld widths are combinations of single weld values multiplied by the parameter obtained from the hollow strut test (×1.25).

## Figures and Tables

**Table 1:** Laser strategies of the strut with inclination of 35.26° (orientation in body centered cubic (BBC) lattice structure).

Strategy/d (mm)	0.5 mm	0.6 mm	0.7 mm	0.8 mm	0.9 mm
**Contour**	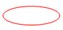	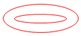	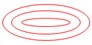	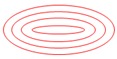	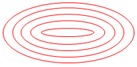
**Standard**	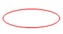	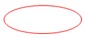	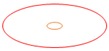	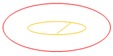	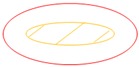

**Table 2:** The porosity level of the strut samples—3D renders with pores shape; the pores in the entire volume were projected to the plane of the view; all images have the same pores scale bar.

	*LP* 225 W	*LP* 250 W	*LP* 300 W	*LP* 350 W	*LP* 400 W
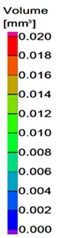	***LS*** 1200 mm/s; ***E_in_*** 7.28 J; ***E_lin_*** 0.19 J/mm	***LS*** 1400 mm/s; ***E_in_*** 9.17 J; ***E_lin_*** 0.18 J/mm	***LS*** 500 mm/s; ***E_in_*** 13.54 J; ***E_lin_*** 0.6 J/mm	***LS*** 500 mm/s; ***E_in_*** 15.43 J; ***E_lin_*** 0.7 J/mm	***LS*** 1700 mm/s; ***E_in_*** 7.56 J; ***E_lin_*** 0.24 J/mm
	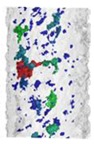	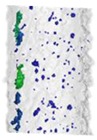	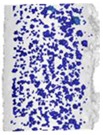	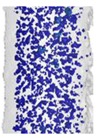	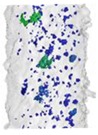
	Por. −0.17%	Por. −0.27%	Por. −1.38%	Por. −2.26%	Por. −0.63%
	***LS*** 700 mm/s; ***E_in_*** 9.02 J; ***E_lin_*** 0.32 J/mm	***LS*** 700 mm/s; ***E_in_*** 9.84 J; ***E_lin_*** 0.36 J/mm	***LS*** 900 mm/s; ***E_in_*** 9.37 J; ***E_lin_*** 0.33 J/mm	***LS*** 1100 mm/s; ***E_in_*** 8.91 J; ***E_lin_*** 0.32 J/mm	***LS*** 900 mm/s; ***E_in_*** 10.17 J; ***E_lin_*** 0.44 J/mm
	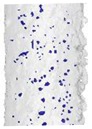	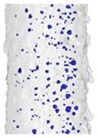	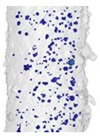	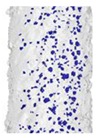	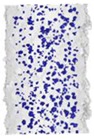
	Por. −0.17%	Por. −0.31%	Por. −0.42%	Por. −0.43%	Por. −0.81%

**Table 3:** Various suitable combination of process parameters for production of the struts with diameters 0.5–0.7 mm, (***N*** (-) is number of the used contour, **w** (µm) is single width × 1.25).

d (mm)	OL (%)	LP (W)	LS (mm/s)	w (µm)	BC (µm)	N (-)	OL in Center (µm)	OL in Center (%)
**0.5**	-	225	600	295	147	1	89	30%
	-	325	1000	293	147	1	86	29%
	-	350	1300	285	143	1	70	25%
	-	375	1200	294	147	1	88	30%
**0.6**	-	400	1000	339	170	1	78	23%
**0.7**	34%	225	900	236	118	2	84	36%
	29%	250	1000	224	112	2	67	30%
